# The Institutional Library

**Published:** 1914-08-08

**Authors:** 


### THE INSTITUTIONAL LIBRARY.

### A Mental Hospital Handbook.

Lunacy Practice: A Practical Guide for the Certification and Detention of Persons of Unsound Mind. By William H. Gattie, of Gray's Inn, Barrister-at-Law. (London: Shaw and Sons. 2s. 6d. net.)

As so many medical men and others have to deal frequently with legal matters in connection with the insane, it is obvious that there must be a demand for such a useful handbook as that compiled by Mr. William H. Gattie. It should be in the hands of all whose duties demand an acquaintance with the various forms which have to be filled up when certification and other matters pertaining to the detention of those of unsound mind have to be carried out. These various forms are reproduced in Mr. Gattie's book, filled in and completed in each case in the manner required; in addition there are many explanatory notes, and also citations of the Lunacy Act bearing upon doubtful points which arise. That such a practical book has not the circulation which it might have may be inferred from the number of errors in filling up forms connected with the detention of the insane which are met with daily. Medical men are very frequent offenders in this respect. Many general practitioners have perhaps not even one case during the year in which certification is required. Consequently when they are called upon to deal with such cases they are frequently at a loss as to the procedure to be carried through; and the queries addressed to the medical officers of mental hospitals and to others whose work makes them familiar with such procedure are therefore numerous. Many of these queries find their answers in Mr. Gattie's book.

### Institutional Segregation.

A History of Penal Methods. By George Ives, M.A. (London: Stanley Paul and Co. Price 10s. 6d. net.)

Institutions and institutional treatment have had so much to do with penal methods that it is not surprising to find Mr. George Ives devoting one chapter of his history to "the treatment of the insane," and yet another to "the visitation of the sick." In the case of the former, punishment and treatment have been extraordinarily interposed. Sometimes because the mentally deranged when wandering abroad, or locked up, as in the case of the wealthier families, at home, made themselves a nuisance, they were punished; sometimes flogging itself was considered a remedy for possession; sometimes those who were entrusted with responsibility for the care of the insane relieved their feelings in this way. Flogging was also found on occasion to subdue their ravings. In 1403 he says the Priory of St. Mary of Bethlem was tending insane patients, and St. Katharine's by the Tower is described in Besant's "London in the Time of the Stuarts" as "used to keep the better sort of mad folks." It was in the middle of the eighteenth century that "grim and sombre" institutions began to be erected for the detention of these unfortunates, described in a quotation from Dr. Conolly's "Treatment of the Insane" as nothing but "prisons of the worst description." Mr. Cullen's medical view was that "fear being the passion that diminishes excitement, may therefore be opposed to the excess of it"; and the result was therapeutic flogging. Then sprang up the "private asylums," which the middle of the nineteenth century exploited in fiction, some capital, some much the reverse, but all the harvest reaped from the abuses of the preceding hundred years. From 1793, when Pinel removed the chains from the patients in the Bicêtre, the modern treatment of insanity may be said to have begun. In 1813 appeared Samuel Tuke's description of the system employed at "The Retreat," which his grandfather had founded at York in 1792, and this largely helped to make known the new and to destroy the old system. In 1813 also were made the allegations against the treatment prevailing at York Asylum, which, when denied by the governors, were taken up by gentlemen in the county who qualified as governors, and brought their votes to bear on the inquiry. The place was burnt down next year, it is said to conceal the guilt of those of the old gang who feared still further disclosures. At Lincoln Asylum, about 1838, mechanical restraints were removed. Concluding this chapter on the treatment of the insane, he says: "Slowly the light of science began to penetrate into the dark places of punishment. The entirely mad were first rescued and treated as patients . . . their case now belongs to medicine, not to criminology."

In dealing with the visitation of the sick Mr. Ives points out that the one real oasis in the prison system was not centred in the chaplain but in the infirmary, "which has been well termed the gaol paradise." He proceeds to show the desperate shifts to which prisoners would go to get into the sick wards, maiming themselves, and even, to oblige a friend, inflicting grave injuries on one another. Thus to certain Prison Commissioners the infirmary itself became suspect, and suggestions were made even for the solitary confinement of the sick. The inspiring fact thus emerges that it was the claims of illness and suffering, and the progress of medical science which accompanied them, which first set the example of humanity to that grim and secret world of tyranny and cruelty of which institutions in the past have been such terrible examples. It is a hard fact of this sort which makes hospital men realise that there is something more than mere sentiment in calling hospital life and nursing life indeed a high calling. Hospitals have been the leaven which has permeated the old system, and one may be grateful to Mr. Ives for his learned and curious researches into the old relations of treatment and punishment in which "the institution" has played such a curious part.

### A Pocket Guide to Nursing.

Outlines of Nursing. By Violet Young. (London: The Scientific Press. Price 1s. net.)

This brief synopsis of elementary points on nursing contains a great deal of useful information packed into a very brief space. From purely internal evidence, we should suspect the authoress has been trained at King's College Hospital, or at least at some school staffed by King's College graduates. For sterilised non-medicated dressings might not exist for all the notice that they here receive; whereas "strong mixture" and other drastic chemical antiseptics, of the type which have been abandoned practically everywhere but at "King's," are fully described. Incidentally, in so condensed a pocket guide as this it seems a pity to include accounts of the manufacturing processes by which antiseptics are produced; indeed, it is arguable whether even in an advanced textbook of nursing these would find a legitimate place. But we should not ourselves attach much antiseptic value to a 1-in-4,000 solution of lysol.

